# PCL/Andiroba Oil (*Carapa guianensis* Aubl.) Hybrid Film for Wound Healing Applications

**DOI:** 10.3390/polym13101591

**Published:** 2021-05-14

**Authors:** Debora F. Silva, Klinsmann T. Lima, Gilmara N. T. Bastos, Johnatt Allan R. Oliveira, Luís Adriano S. do Nascimento, Carlos Emmerson F. Costa, Geraldo N. R. Filho, Viktor O. C. Concha, Marcele F. Passos

**Affiliations:** 1 Laboratory of Oils of the Amazon, Federal University of Pará, Belém 66075-750, PA, Brazil; dfsdeborafreitas@gmail.com (D.F.S.); adrlui1@yahoo.com.br (L.A.S.d.N.); emmerson@ufpa.br (C.E.F.C.); geraldonrf@gmail.com (G.N.R.F.); 2 Laboratory of Neuroinflammation, Federal University of Pará, Belém 66075-110, PA, Brazil; klinsmanntl@gmail.com (K.T.L.); bastosgnt@gmail.com (G.N.T.B.); 3 Department of Nutrition, Federal University of Pará, Belém 66073-040, PA, Brazil; johnattrocha@yahoo.com.br; 4 Department of Chemical Engineering, Federal University of São Paulo, Diadema 09913-030, SP, Brazil; viktor.cardenas.c@gmail.com

**Keywords:** polycaprolactone, wound healing, andiroba oil, polymer, cell viability

## Abstract

Developing a biomimetic material to wound care is an emerging need for the healing process. Poly (ε-caprolactone) (PCL) is a polymer with the necessary dressing’s requirements often used in medicine. Their surface, physic-chemical and biological properties can be modified by adding bioactive compounds, such as andiroba seed oil (*Carapa guianensis*). This Amazonian natural plant has medicinal and pharmacological properties. For this purpose, PCL polymeric films incorporated with andiroba oil were investigated. The synthesis of hybrids materials was carried out in the solvent casting method. Thermal properties were evaluated using thermogravimetric analysis (TGA/DTGA) and differential scanning calorimetry (DSC). The solvent type on the surface and hydrophilicity of samples was studied using a scanning electron microscope (SEM). Additionally, contact angle measurements, functional groups analysis, fluid absorption capacity, and cell viability were performed. The results demonstrated the influences of andiroba oil under the morphology and thermal properties of the polymeric matrix; the hydrophilicity of the hybrid film obtained by acetic acid was reduced by 13%; the porosity decreased as the concentration of oil increased, but its higher thermal stability. The L929 cell line’s proliferation was observed in all materials, and it presented nontoxic nature. It was demonstrated the ability of PCL hybrid film as a matrix for cell growth. Then, the materials were proved potential candidates for biomedical applications.

## 1. Introduction

Researchers have aroused interest in using biomimetic dressings, with bioactive properties and as carriers of medicines, mainly for exudative wounds or resulting from prolonged pressure on the skin [1,2]. These sterile therapeutics covers may interact and imitate harmful tissue, reducing the time for treating the lesion, with cell regeneration and proliferation in the affected region; they have adequate porosity and fluid absorption capacity. Dressings must also avoid complications for the patient, differentiating themselves in physical and chemical terms to meet different biological and mechanical needs. Thus, they need to be easy to apply and remove; they must have nontoxic, nonallergenic, and biocompatible properties.

Hydrogels and silicones are commonly used as smart dressings for exudative wounds due to the high potential for swelling [3,4]. However, they have been causing inflammation and harmful monomers’ release during treatment [5,6]. Thus, the search for bioactive materials with good biological properties and low-cost becomes essential. Moreover, technological advances in wound dressings are no longer restricted to producing materials with static purposes of mechanical protection and gas exchange through the membrane. The transport of therapeutic compounds such as hormones, anticancer agents, drugs, and other assets has enabled patients to be more independent and practical [7,8,9,10].

Poly (ε-caprolactone) (PCL) has been applied in conjunction with other additives for use in biomedical applications. It is an acyclic polyester with excellent elastic, biodegradable, and bioabsorbable properties [11]. The Food and Drug Administration has approved PCL to use in drug delivery devices and various structures as nanospheres, nanofibers, foams, meshes, membranes, and scaffolds [12,13]. Furthermore, as a temporary substitute (scaffold) of the cell-matrix, the PCL allows cell adhesion and stimulates new ones’ growth [14]. Muwaffak et al. [15] produced polycaprolactone filaments interspersed with antimicrobial metals using a hot-melt extruder and 3D printing techniques, obtaining potent antibacterial dressings. Poly (ε-caprolactone) nanofibers coated with chitosan and gamma oryzanol also showed results for wound healing in mice [16]. On the other hand, incorporating vegetable oils in hybrid nanocarriers has decreased cytotoxicological effects and increased the dressing’s antimicrobial properties [17]. Cardoso et al. [18] evaluated the effect on molar mass distribution of jojoba and andiroba oil encapsulation by miniemulsion polymerization. Uscátegui et al. [19] studied the cytotoxicity and antibacterial activity of polyurethanes based on modified castor oil and polycaprolactone. However, few studies on the intercalation of vegetable oils under alpha-hydroxy ester polymeric matrices and their potential for wound healing have been discussed in the literature via solvent casting technique. The possibility of adding bioactive natural compounds to PCL molecular chains represents a springboard to produce new dressings with different therapeutic qualities and low-cost.

Several oils and extracts of medicinal plants and dressings of animal origin [20] are currently used in regenerative medicine to stop bleeding, to reduce the effects of contamination on injuries, to modulate the healing process, and to promote better esthetic results with reduced scar formation [21,22]. The andiroba (*Carapa guianensis*) seed oil is widely used in traditional medicine for its pharmacological properties. It is extracted from the seed and has anti-inflammatory, analgesic, antiallergic, antimicrobial, and healing properties, which are derived from its biologically active constituents such as limonoids and triterpenes [23,24,25]. Thus, the present study aimed to develop PCL films incorporated with andiroba oil (hybrids) like a scaffold to the wound healing process, using the solvent casting technique. This technique is commonly used to synthesize PCL, and PCL-based films for wound dressings purposes, where porous and bio-resorbable materials have been obtained in an accessible manner [26]. It does not require specific equipment and is suitable for the large-scale production of low cytotoxicity and cost wound dressings [27]. Here, we focus on PCL’s hybridization, using andiroba oil as an active compound to create a multifunctional wound dressing. The objective was to assess these materials’ thermal, surface, and biological properties, to a more natural and straightforward treatment.

## 2. Materials and Methods

### 2.1. Materials

Poly (ε-caprolactone) (PCL) (M_n_ = 45,000 and δ = ((9.65 cal cm^−3^)^1/2^)), dimethyl sulfoxide, phosphate-buffered saline (PBS) and amphotericin B were supplied by Sigma-Aldrich (Barueri, Brazil). Andiroba oil (AO) was provided by Amazon Oil (Belém, Brazil). Its physic-chemical characteristics are listed in Table 1. Acetone ((δ* = 9.77 (cal cm^−3^)^1/2^), acetic acid ((δ* = 10.5 cal cm^−3^)^1/2^) and dichloromethane (δ* = 9.70 (cal cm^−3^)^1/2^) were purchased from Neon Comercial (Belém, Brazil). Penicillin-streptomycin was supplied by Nacalai Tesque (Kyoto, Japan). The L929 cell line was obtained from American Type Culture Collection (Rockville, MD, USA). *δ = solubility parameter [28,29,30].

### 2.2. Fabrication of PCL Casting Films

**Figure 1:**
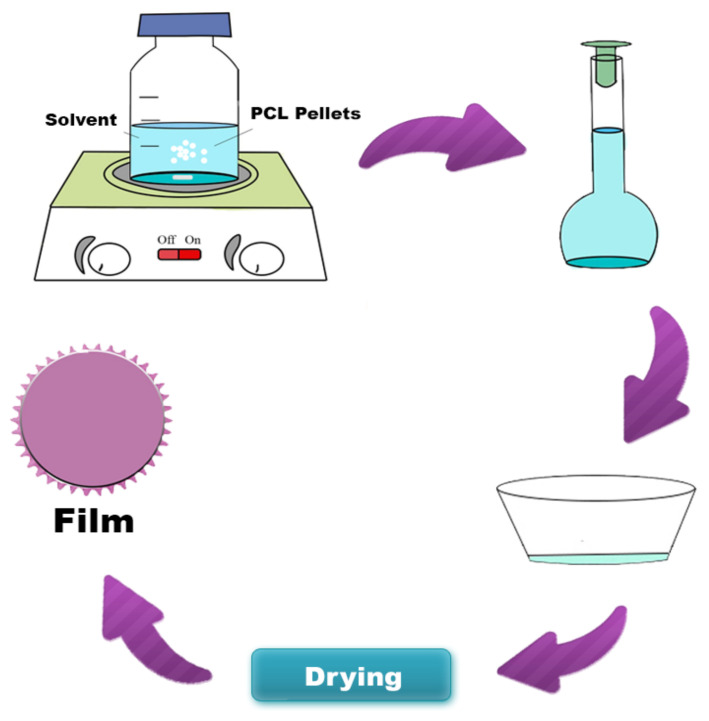
Schematic diagram of poly (ε-caprolactone) (PCL) film synthesis by the solvent casting technique.

PCL hybrids films were obtained from the solvent casting technique (Figure 1), using different solvents (acetic acid, acetone, and dichloromethane). The polymer solution concentration was kept constant at 5% *w/v*. Andiroba oil was added to the polymeric solution at room temperature until homogenized, without phase separation. Two concentrations were evaluated to hybrids films, 1.7% *w/w* and 2.7% *w/w* (g of andiroba oil/g of PCL). After 10 min stirring, the mixture (PCL solution/oil) was applied to the cylindrical silicone mold, with a volume constant (5 mL). The films were dried for one week at room temperature and another four days in a vacuum oven at 40 °C to remove the solvent’s residual traces. PCL without oil was synthesized as a control film under the same conditions. Table 2 summarizes the compositions of each sample.

### 2.3. Fatty Acid Composition of Andiroba Oil (Carapa guianensis)

The fatty acid composition of andiroba oil was determined by the American Oil Chemists’ Society’s official methods [31,32]. It was used a Varian CP 3800 gas chromatograph system (GC) (São Paulo, Brazil), equipped with a capillary column Agilent (30 m × 0.32 mm CP WAX 52 CB) (Santa Clara, CA, USA) and a hydrogen flame ionization detector (Varian, São Paulo, Brazil). The analysis was performed under the following conditions: helium as a carrier gas, adjusted at a flow rate of 1 mL min^−1^; programmed temperature to ramp from 50–250 °C at 10 °C. min^−1^; detector temperature set to 270 °C; split mode injection for 1 µL (1:10); and injector temperature at 250 °C. The quantitative data were performed by peak area normalization. The fatty acid methyl esters were identified by comparing their retention indices with fatty acid methyl ester standards (Table 3). The analyses were performed in triplicate.

### 2.4. Thermal Analysis

A Shimadzu DTG-60H thermogravimetric analyzer (TGA) (Kyoto, Japan) was used to evaluate samples’ thermal stability. Approximately 5–8 mg of samples were weighed into an aluminum pan. The experiments were done from 25 to 550 °C at a heating rate of 10 °C. min^−1^, under dynamic nitrogen atmosphere (50 mL min^−1^). The glass transition temperature (*T_g_*) and melting temperature (*T_m_*) were determined using a differential scanning calorimeter, model DSC60 plus (Kyoto, Japan, Shimadzu Corporation). The specimens were scanned from 35 to 250 °C, with isotherms for 5 min, cooling from 250 to 35 °C, isothermal for 5 min, and heated again from 35 to 250 °C. The films’ thermal history was eliminated in heating and cooling consecutive programs, and the second run measured the transition temperatures. TGA and DSC thermograms were evaluated by Origin pro 9.1 software (Originlab Corporation, Northampton, MA, USA). Enthalpy of fusion (*ΔH_m_*) was determined by the area under the endothermic peak (integrating the melting peak below the baseline). The degree of crystallinity (*xc*) of films was calculated using Equation (1)

(1) $$xc\;\left(\%\right)=\frac{\Delta Hm}{\Delta Hm^{\infty }\varphi }\;\times \;100$$

where Δ*Hm*, Δ*Hm*^∞^, and *φ* are the experimental endothermic enthalpy of fusion (J g^−1^), the theoretical enthalpy of 100% crystalline PCL, taken 136.5 J g^−1^ [33] and the weight fraction of the PCL.

### 2.5. Fourier-Transform Infrared Spectroscopy (FTIR)

FTIR absorption spectra were recorded using a Agilent spectrophotometer, model cary 630 (Hanover, Germany), equipped with ATR to evaluate specimens’ vibrational mode. IR analysis was carried out in the range from 4000 to 650 cm^−1^, with a resolution of 8 cm^−1^ and 32 scans.

### 2.6. Surface Characterization

The surface morphology (porosity and roughness) was investigated using a Jeol JSM-6610LV scanning electron microscope (SEM) (Tokyo, Japan). The samples were covered with a thin gold layer (Denton Vacuum, model Desk V, Moorestown, NJ, USA) and evaluated with accelerating voltage 1 kV and magnification 1000×. The hydrophilicity property of polymer film was investigated using angle contact angle measurements. The sessile drop method was used at room temperature. The average angle of deionized water droplets on the material’s surface was obtained from six measurements for each sample. Water drop images were recorded using Nikon b500 16 mp/40× photographic camera and analyzed by Image J. software. The thickness of the pieces was determined using the micrometer.

### 2.7. In Vitro Tests

#### 2.7.1. 3D Cell Culture

Fibroblast lineage cells (L929) (Rockville, MD, USA) were incubated in 96-well plates at a density of 2 × 10^4^ cells/well. The experiment was performed in an incubator at 37 °C, humidified, 5% CO_2_/95% air environment at 24 h. The Dulbecco’s modified Eagle’s medium (DMEM) with 10% bovine fetal serum (SBF)(Gibco^TM^, Sao Paulo, Brazil), contained 100 units/mL penicillin, and 50 μg/mL streptomycin was used for cell nutrition and maintenance.

#### 2.7.2. Cell Viability Assay

The films were placed at the bottom of the well covering all surfaces, and cell viability was performed using the 3-(4,5-dimethyl-thiazol-2-yl)-2,5-diphenyl-tetrazolium bromide (MTT) assay [34]. The methodology consists of soluble chromogen (MTT) conversion to slightly soluble formazan via dehydrogenases present in viable cells. Then, dimethyl sulfoxide (100 ul) was added to each well to measure the MTT product (formazan). After, the content of formazan crystals was evaluated at 570 nm by a scanning multi-well spectrophotometer plate reader (ELISA reader, Bio-Rad 2550 EIA, Hercules, CA, USA). The blue color intensity in control (only cells) wells was designated as 100% viability. The groups evaluated were: (1) control (cell only); (2) PAcA-control; (3) PAcA-1.7, and (4) PAcA-2.7. All further comparisons were based on this reference level to determine the percentage of cell viability. The experiments were performed in quadruplicate, and the results are presented as mean ± standard deviation.

#### 2.7.3. Fluid Absorption Capacity/Degree of Swelling

The phosphate-buffered saline (PBS) and protein milk-based solution, both with pH = 6.3, were used for the assessment of biomaterial as a function of macromolecule permeability. The materials were previously cut into pieces of 1 cm^2^ and weighted. Through the differences in the weight of the materials, the absorption volume was quantified by Equation (2). *W_s_* is the swollen sample weight, and *W_d_* is the dry sample weight. The immersion was done at 37 °C (body temperature) for 1 h.

(2) $$Degree\;of\;swelling\;\left(\%\right)=\frac{Ws-Wd}{Wd}\;\times 100$$

#### 2.7.4. Statistical Analysis

The significance of the difference between the mean concentration of material absorption and standard control was tested using the Mann-Whitney U procedure for nonparametric data sets. The meaning of cell viability was performed by ANOVA One-Way, with a significance level of *p* < 0.05. The GraphPad Prism software (version 6.01, La Jolla, San Diego, CA, USA) was used.

## 3. Results and Discussion

### 3.1. Fatty Acid Composition of Andiroba Seed Oil (Carapa guianensis)

Andiroba oil (AO) has often been studied as an insect repellent [35]. However, their physic-chemical properties can offer new opportunities for use in wound care. Table 3 shows the chemical composition of the fatty acids in the andiroba seed oil, and the profile agrees with that described in the literature [36,37]. In total, twelve methyl esters were identified, and about 60.5% account for the averaged composition of unsaturated fatty acids. The major compounds found were oleic acid (48.67%), palmitic acid (26.89%), linoleic acid (10.79%), and stearic acid (8.80%). In smaller percentage following acids can be seen: lauric (0.89%), myristic (0.68%), palmitoleic (0.81%), arachidic (1.30%), behenic (0.25%), heptadecanoic (0.29%) and lignoceric acid (0.20%). These acids play a key role in wound care, with emollient action and biological properties [37,38]. Cutaneous dressings need to act as a bacterial barrier and prevent dehydration of the skin. Linoleic acid integrates the stratum corneum and prevents transepidermal water loss, ensuring its integrity [39,40]. This compound favors autolytic debridement in the wound and accelerates the healing process.

Additionally, oleic acid (Omega 9) and linoleic acid (Omega 6) are essentials fatty acids (AGE), which participate in human metabolism and nutrition. AGE induces the tissue granulation process, facilitates cell proliferation, and increases the membrane’s cell permeability, protecting the lesion [40]. The presence of non-fatty acid components (tocopherols, carotene, and tetranortriterpenoids) also attributes other medicinal properties and anti-inflammatory effects to the AO [37,41,42,43].

### 3.2. Surface Characterization

#### 3.2.1. Optical and SEM Imaging of PCL Casting Films

**Figure 2:**
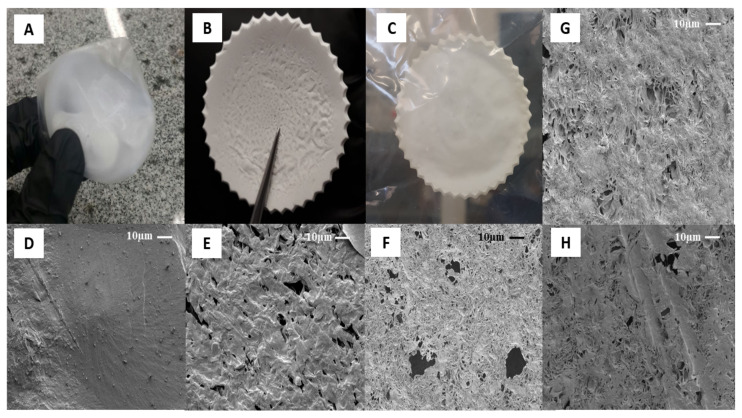
The optical images (OI) and microscopic (SEM) of PCL casting film at magnification 1000×, respectively: (**A**) PD-control OI; (**B**) PAC-control OI; (**C**) PAcA-control OI; (**D**) PD-control SEM; (**E**) PAC-control SEM; (**F**) PAcA-control SEM; (**G**) PAcA-1.7 SEM; (**H**) PAcA-2.7 SEM.

The type of solvent and their evaporation rate are some critical parameters that affect the surface, physic-chemical and biological properties of films on the solvent casting method [44]. Figure 2 shows the optical and microscopic images of PCL control and hybrids film, respectively. Three different solvents were evaluated: acetone, dichloromethane, and acetic acid to determine an optimum solvent system. All polymer solutions formed opaque films. No macroscopic phase separation was observed after casting of samples, which suggests high PCL molecular weight and enough Van der Waals forces to aggregate the solution’s polymer chains [45]. Figure 2A shows optical images of samples dissolved in dichloromethane (DCM). The high solvent evaporation rate (boiling point 39.6 °C) induced specimens with body deformation, air bubbles, inhomogeneous surfaces, irregular shape, and dimensional shrinkage [44]. The solvent molecules diffusion fast generated smooth and dense surfaces, as can be seen in Figure 2D. Although nonpolar organic solvent facilitates the PCL solubilization process due to higher solvent-polymer chemical affinity, the low difference between its solubility parameters ((δ = 0.05 (cal cm^−3^)^½^) can make difficult the mass transport of volatile and the use of DCM as a porogen [46].

On the other hand, porous structures were observed on films formed by acetone (boiling point 56.0 °C, Figure 2E) and acetic acid (boiling point 118 °C, Figure 2F). Volatile species (solvent) escaped from the film surface and allowed pores’ organization to different diameters (0.5 to 19.8 μm). This characteristic plays a role in inducing cell penetration and proliferation, the mass transport of nutrients, and tissue growth [47]. Additionally, acetone vapor trapping under solidified samples was observed (central region, Figure 2B). Factors as room temperature, drying environment, humidity [48] and Amazonian climate affected the solvent’s gradual evaporation kinetics (low evaporation rate). Acetic acid was observed to possess excellent film-forming properties. The samples presented a regular surface, uniform thickness, and good mechanical handling strength (Figure 2C). This behavior may have been influenced by viscoelasticity, the degree of entanglement of the chains in solution, and the solvent molecules’ size [49]. An acidic solvent can decrease the PCL polymeric chain by breaking of ester linkages, affecting the viscosity of solution and morphology on the surface [50]. Considering its potential to inhibit bacteria colonization, such as *Escherichia coli* and *Staphylococcus aureus* [45], the acetic acid was selected as the optimum solvent system to obtain PCL hybrids films (PCL/AO). By adding AO, in the case of samples PAcA-1.7 (Figure 2G) and PAcA-2.7 (Figure 2H), a decrease in the number and size of pores was observed. From the SEM micrograph, AO partially filled the empty spaces on the material surface. The porosity decreases as the concentration of oil increases. Visually, there are no significant differences between the oil-loaded film and the control sample; only an increase in the film’s initial thickness was detected (>50%). No migration of oil to the surface was observed, suggesting good interaction to system PCL/AO.

#### 3.2.2. Water Contact Angle Measurement

Table 4 displays the water contact angle measurements for control and hybridized polycaprolactone film, and the results were reported as mean values and standard deviation. Andiroba oil content and surface morphology affected the hydrophilic property of samples, decreasing their interaction with water. This behavior can be explained by the nonpolar group content on the film’s surface and triglycerides’ chemical structure. The contact angle values were observed as 80.26° (PAcA-control), 87.25° (PAcA-1.7), and 91.39° (PAcA-2.7), and are according appropriate range for adhesion cell of scaffolds [51,52].

### 3.3. Fourier-Transform Infrared Spectroscopy (FTIR) Analysis

**Figure 3:**
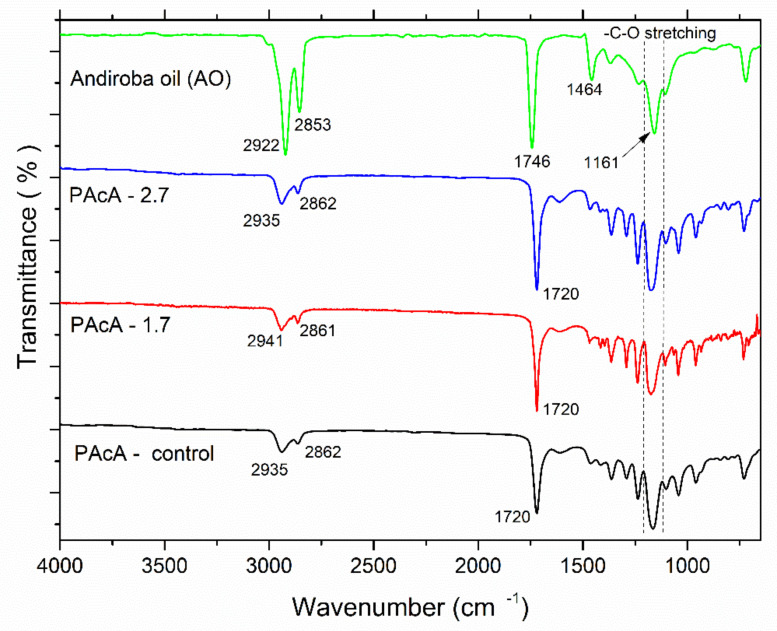
Fourier-transform infrared spectroscopy (FTIR) of andiroba oil and control and hybrids film of PCL.

FTIR spectroscopy is a useful technique to investigate the chemical structure of organic molecules and understanding changes in their band position and vibrational modes. Clearly, the IR visual inspection showed great similarities and overlapping of the data in samples’ spectral profiles (Figure 3). In the spectrum of andiroba oil, the fingerprint region at 2900–2850 cm^−1^ is assigned to stretching vibration of CH_2_ groups; peaks at 1746 cm^−1^ and 1464 cm^−1^ are attributed to -C=O stretching (saturated aliphatic ester) and -C-H bending vibrations, respectively; bands in the range 1200–1160 cm^−1^ are related to the—C-O stretching [35,53]. PCL typical bands were observed for all casting films: in the region 3000–2860 cm^−1^, which represents the C-H asymmetric and symmetric stretching vibration of aliphatic carbons and peaks at 1720 cm^−1^, due to the presence of the ester carbonyl group (C=O stretching) [54,55,56,57]. Some differences in the peak intensity and position also may be observed for hybrid samples (PAcA-1.7 and PAcA-2.7), according to the increasing amount of andiroba oil. Bands at 1160 and 1165 cm^−1^ are assigned to -C-O stretching of andiroba oil [53] and to the -C-O stretching vibration in the amorphous phase of PCL [57].

### 3.4. Thermogravimetric Analysis and Its Derivates (TGA and DrTGA)

**Figure 4:**
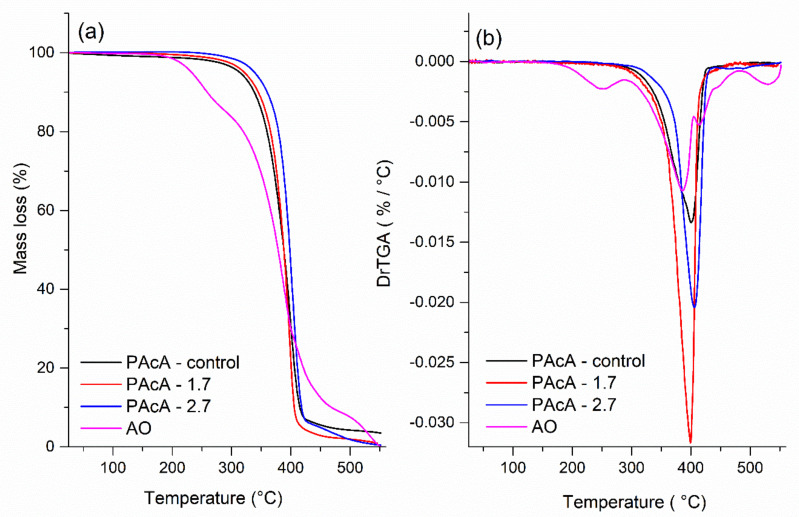
(**a**) TGA (**b**) and DrTGA analysis of andiroba oil and PCL films in acetic acid (PAcA-control, PAcA-1.7, PAcA-2.7).

Figure 4 displays the TGA curves and their derivates (DrTGA) for PCL control and hybrid film and andiroba oil. The maximum decomposition (Tdmax), onset degradation temperatures (Tonset), mass loss, and the percentage of residues are given in Table 5. The thermal degradation of the PAcA-control sample occurs between 242.7 °C (Tonset) and 450 °C, in a single mass loss step (96.5%) and maximum degradation temperature (Tdmax) of 249.8 °C. This event can be attributed to the chemical bond cleavage of polyester chains by the pyrolysis reaction. In Figure 4a, it was observed four stages of weight loss for andiroba oil (AO), which began to degrade at 150 °C (Tonset) and are entirely decomposed above 531.3 °C with 99.9% of weight loss. The first step can be attributed to the evaporation and/or pyrolysis of smaller carbon chain fatty acids (C12—C16:1) at the maximum degradation temperature (Tdmax) of 249.8 °C. Triglycerides with long-carbon chains tend to be thermally decomposed above 288.1 °C (Tonset) due to the higher boiling point. Around 404 °C and 482 °C (Tonset) probably occur the decomposition of stearic, oleic, linoleic, linolenic fatty acids, and others [38]. From Figure 4b, we also observed that incorporating higher levels of andiroba oil in PCL film tends to shift the Tonset and Tdmax to higher values and reduces the mass residue percentage (0.5%). This increase in the degradation temperatures may be related to the barrier effect caused by polymer chains to the AO molecules, making it difficult to diffusion volatile compounds and increase the thermal stability of hybrid materials.

### 3.5. Differential Scanning Calorimetry (DSC) Nonisothermal Studies

**Figure 5:**
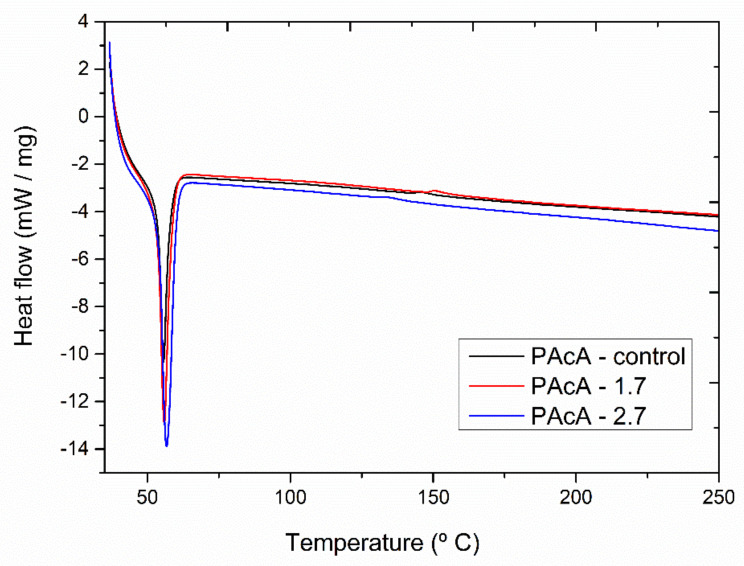
DSC (second heat) analysis of andiroba oil and PCL films in acetic acid (PAcA-control, PAcA-1.7, PAcA-2.7).

Figure 5 shows the DSC thermograms for PCL control and hybrid samples. All materials revealed an endothermic peak between 56 and 58 °C, attributed to the polymeric matrix melting. The oil incorporation did not show a significant shift on Tm peak. However, more porous media (PAcA-control) can provide higher surface area contact and reduce the flow resistance, improving the heat transfer of scaffolds [58,59].

The melting enthalpy (areas of endothermic peaks) for the samples PAcA-control, PAcA-1.7, and PAcA-2.7 was 38.93 J g^−1^, 46.70 J g^−1^, and 61.31 J g^−1^ (Table 6). An increase in the degree of crystallinity (28.52 to 44.91) and Δ*H* was also observed as the oil content increases, decreasing the amorphous domain. The PAcA-2.7 sample showed an increase of 57.46% to the degree of crystallinity about PAcA-control. The oil dispersion within the PCL chain made it difficult to evaporate the solvent via casting technique, decreasing the evaporation rate of acetic acid. Thus, the polymer chains had more time to reorganize into a more crystalline conformation. Gomes [60] produced more crystalline PCL fibers, reducing the acetic acid content, using 95:5 acetic acid:water ratios. The low volatility and low chemical affinity of acetic acid with the PCL’s hydrophobic chain lead to less aggressive solubilization, affecting crystal domains’ formation [61,62].

### 3.6. Cell Viability Assays (MTT)

**Figure 6:**
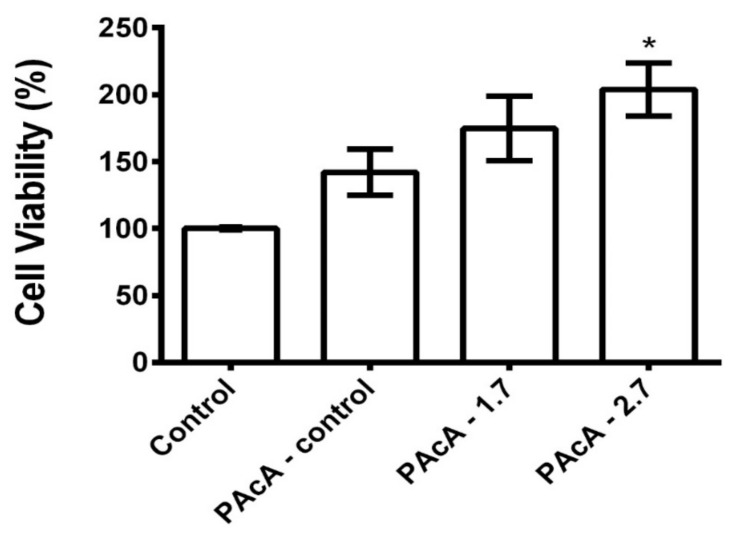
Cell viability of L929 cells on PAcA-control and hybrids (PAcA 1.7 and PAcA-2.7) film after 24 h in culture. *p* < 0.05, n = 4. The asterisk (*) indicates a significant difference between the control and treated groups.

As shown in Figure 6, the PAcA-control and hybrids film did not demonstrate cell death 24 h after cell seed. L929 cells were grown on PAcA-control film, and PAcA-1.7 and PAcA-2.7 samples had a viability of 142.0 ± 29.85, 174.8 ± 41.72, and 203.8 ± 34.33, respectively. However, there was no statistical difference (*p* < 0.05) between the PAcA-control and hybrids compared to the control group (cell only). In all samples, the proliferation of L929 cells was observed, indicating the high-affinity of the cells for the polymer. Our results are following cytotoxic effects to biodegradable polyester films [63,64]. Both the PAcA-control polymer and the polymer incorporated with the andiroba oil (hybrids sample) do not induce a toxic response on the L929 cell line, demonstrating the ability of PCL casting film for cell viability and growth. The insertion of andiroba oil in the PCL matrix also helps in the high cell viability presented. The oil has anti-inflammatory, antimicrobial, and antiallergic potential effects [25], is widely used in traditional medicine to treat joint pain and treat muscle and skin injuries [65,66]. The oil still may have antioxidant activities that are important during the inflammatory phase of the healing process [67].

### 3.7. Biological Fluid Absorption Capacity

**Figure 7:**
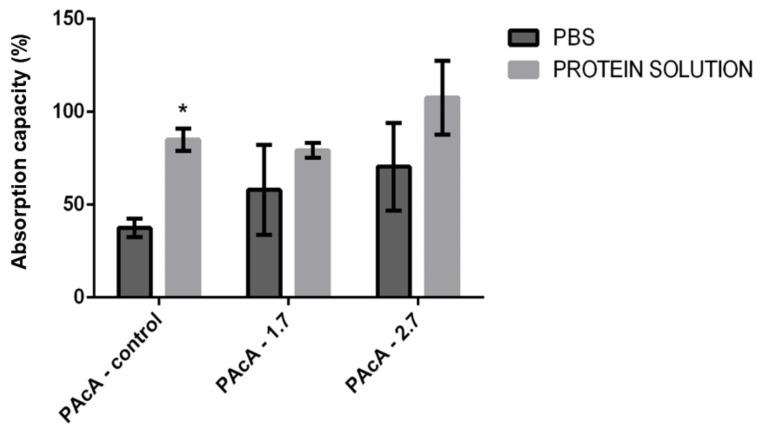
Evaluation of the fluid absorption capacity of PCL control polymeric films (PAcA-control) and incorporated with andiroba oil (PAcA-1.7 and PAcA-2.7) immersed for one hour in aqueous or protein solution. The data were plotted in the average format and standard error of the mean. The analysis was performed by 2-way ANOVA followed by Bonferroni’s test, with *p* < 0.05, n = 4. The asterisk (*) indicates a significant difference between the control and treated groups.

Dressings that assist in absorbing exudate, preventing infection, covering the wound, and promoting a moist environment, consequently improve the conditions of the wound bed and assist in its healing [68,69]. We evaluated the material’s degree of swelling in different test solutions to use the material as wound dressings, whether exudative or not. Our results suggest a statistically significant difference only between the group with the PAcA-control film, in which greater permeability of protein solution (milk) was observed compared to an aqueous solution (PBS). The swelling may have been influenced by the chemical interactions between solution and hybrid films, molecular diffusion, and surface characteristics. There was no statistically significant difference between the PAcA-1.7 and PAcA-2.7 (Figure 7).

## 4. Conclusions

In this study, PCL-based hybrid films were prepared by solvent casting technique for wound healing applications. Andiroba-oil-loaded PCL membranes were evaluated by surface morphology, water contact angle, functional groups, and thermal behavior. FTIR showed great similarities and overlapping of the data in samples’ spectral profiles. Water contact angle measurement and SEM images showed that the AO affects samples’ hydrophilicity and porosity, decreasing their interaction with water. DSC analyses showed that andiroba oil affects the evaporation rate of solvent and increases the crystals domains. TGA analysis suggested higher thermal stability of hybrid materials due to the barrier effect caused by polymer chains to the AO molecules. Viability tests demonstrated the absence of cytotoxicity and proliferation of L929 cells on PCL—AO film. Moreover, the absorption capacity presented by the material probably can make it suitable for biomedical applications, such as wound dressings, which can assist in covering, preventing infection, and, consequently, in the wound healing process. However, mechanical properties assays, antimicrobial and antioxidant activity, and degradation tests in the future should be performed for a successful application of scaffolds in medicine regenerative.

## Figures and Tables

**Table 1:** Physicochemical properties of commercial andiroba oil.

Physicochemical Data	Units	Values *
Acidity level	% weight	<15.0
Density	25 °C g/mL	0.9261
Iodine index	gI_2_/100 g	55–80
Melting point	°C	22
Peroxide content	10 meq O_2/_kg	<10.0
Saponification index	mg KOH/g	190–210
Unsaponifiable matter (bioactive)	%	3–5

* Data obtained by the Amazon Oil supplier.

**Table 2:** Samples identification and composition.

Sample Name	Solvent	Andiroba oil (AO) (% wt _OIL_/wt _PCL_)
PAcA-control	Acetic Acid	-
PAcA-1.7	Acetic Acid	1.7
PAcA-2.7	Acetic Acid	2.7
PAC-control	Acetone	-
PAC-1.7	Acetone	1.7
PAC-2.7	Acetone	2.7
PD-control	Dichloromethane	-
PD-1.7	Dichloromethane	1.7
PD-2.7	Dichloromethane	2.7

**Table 3:** Fatty acids composition of andiroba seed oil (*Carapa guianensis*) determined by gas chromatography.

Nomenclature	Chain	Composition * (%)
Lauric acid	C12:0	0.89 ± 0.38
Myristic acid	C14:0	0.68 ± 0.25
Palmitic acid	C16:0	26.89 ± 0.98
Palmitoleic acid	C16:1	0.81 ± 0.06
Heptadecanoic acid	C17:0	0.29 ± 0.34
Stearic acid	C18:0	8.80 ± 0.10
Oleic acid	C18:1 (ω-9)	48.67 ± 1.19
Linoleic acid	C18:2 (ω-6)	10.79 ± 0.39
Linolenic acid	C18:3 (ω-3)	0.22 ± 0.02
Arachidic acid	C 20:0	1.30 ± 0.16
Behenic acid	C22:0	0.25 ± 0.14
Lignoceric acid	C24:0	0.20 ± 0.04

* Average values and standard derivations of the three andiroba oil (AO) samples studied.

**Table 4:** Water contact angle measurements (average ± standard deviation) for control and hybrid samples of PCL.

Samples	Water Contact Angle/Degrees
PAcA-control	80.26 ± 5.72
PAcA-1.7	87.25 ± 6.05
PAcA-2.7	91.39 ± 5.10

**Table 5:** TGA and DrTGA curves parameters of the PCL casting films and andiroba oil: degradation onset temperature (Tonset), maximum degradation temperature (Tdmax), weight loss (%), and the percentage of residues.

Samples	Tonset (°C)				Tdmax (°C)				Weight Loss (%)	Residue (%)
	1° Stage	2° Stage	3° Stage	4° Stage	1° Stage	2° Stage	3° Stage	4° Stage		
PAcA-control	242.7	-	-	-	402.4	-	-	-	96.5	3.5
PAcA-1.7	262.9	-	-	-	401.5	-	-	-	99.1	0.9
PAcA-2.7	269.9	-	-	-	408.9	-	-	-	99.5	0.5
AO	150.0	288.1	404.0	482.0	249.8	386.0	413.5	531.3	99.9	0.1

**Table 6:** DSC curves parameters of the PCL casting films: onset melting temperature (Tm onset), peak melting temperature (Tm peak), enthalpy for melting (ΔH), and degree of crystallinity (xc%).

Parameters				
Samples	Tm Onset (°C)	Tm Peak (°C)	ΔH (J g^−1^)	xc (%)
PAcA-control	54.40	56.54	38.93	28.52
PAcA-1.7	54.23	57.22	46.70	34.21
PAcA-2.7	54.15	58.79	61.31	44.91
